# Chegou a Hora de uma Nova Terapia Padrão para a Insuficiência Cardíaca com Fração de Ejeção Reduzida?

**DOI:** 10.36660/abc.20200983

**Published:** 2021-07-15

**Authors:** Eduardo Thadeu de Oliveira Correia, Letícia Mara dos Santos Barbetta, Evandro Tinoco Mesquita

**Affiliations:** 1 Hospital Universitário Antônio Pedro NiteróiRJ Brasil Hospital Universitário Antônio Pedro,Niterói, RJ - Brasil; 2 Complexo Hospitalar de Niterói NiteróiRJ Brasil Complexo Hospitalar de Niterói,Niterói, RJ - Brasil

**Keywords:** Insuficiência Cardíaca, Volume Sistólico, Tratamento Farmacológico, Mortalidade, Hospitalização, Remodelação Ventricular, Neprilisina, Angiotensinas

## Introdução

A insuficiência cardíaca (IC) com fração de ejeção reduzida (ICFER) leva à mortalidade, piora da qualidade de vida, e tem um impacto grande no sistema de saúde. Apesar de ensaios mostrarem a superioridade dos benefícios do inibidor da neprilisina e do receptor da angiotensina (INRA) em relação aos inibidores de enzima conversora da angiotensina (IECA), e a superioridade dos inibidores do cotransportador sódio-glicose 2 (SGLT2i) em relação ao placebo, as diretrizes atuais ainda recomendam o uso de IECA, antagonistas de receptor mineralocorticoide (ARM), e betabloqueadores como primeira linha de terapia da ICFER.^1 - 3^ Nesta carta, serão discutidos os possíveis benefícios e riscos de se adotar INRA e SGLT2i como primeira linha de terapia da ICFER.

## Quais são os possíveis benefícios e riscos de se adotar INRA como primeira linha de terapia da ICFER?

No estudo PARADIGM-HF, os pacientes de ICFER tratados com INRA tiveram redução significativa do principal resultado de mortalidade cardiovascular ou hospitalização por IC (21,8% vs. 26,5%; número necessário para tratar (NNT) = 21) em comparação ao enalapril.^1^ Além disso, o INRA diminuiu significativa a mortalidade global (17,0% vs. 19,8%; NNT = 36).^1^Uma subanálise do estudo PARADIGM-HF também demonstrou que o INRA melhorou significativamente a qualidade de vida em comparação com o enalapril.^4^ Em relação à segurança, o sacubitril/valsartana levou a proporções mais altas de hipotensão e angioedema não grave, mas a proporções mais baixas de insuficiência renal, hipercalemia, e tosse, em comparação com o enalapril.^1^ A superioridade dos benefícios do INRA em relação ao enalapril também foi confirmada em pacientes com ICFER hospitalizados por IC aguda descompensada, no estudo PIONEER-HF, que demonstrou uma redução significativa do peptídeo natriurético tipo B N-terminal (NT-proBNP) em pacientes tratados com INRA desde a semana 1.^5^ A redução de uma combinação de re-hospitalização por IC ou morte cardiovascular também mostrou ser significativa em pacientes tratados com INRA em uma análise exploratória do estudo PIONEER-HF.^6^ Por último, uma subanálise do estudo PIONEER demonstrou que o INRA era bem tolerado e superior ao enalapril para a melhoria dos resultados clínicos independentemente do inibidor ECA, tratamento com BRA, ou de histórico anterior de IC.^7^

Dados reais do registro *Change the Management of Patients with Heart Failure* (CHAMP-HF - Alteração da Gestão de Pacientes com Insuficiência Cardíaca) também demonstraram uma associação entre o tratamento com INRA e melhorias precoces no status de saúde, em comparação com pacientes não tratados com INRA.^8^ No estudo EVALUATE-HF, que tinha o objetivo de avaliar se o INRA, em comparação com o enalapril, melhorou a rigidez aórtica central e remodelação cardíaca, o INRA levou a uma redução significativa de desfecho ecocardiográfico secundário, sugerindo que o INRA possa induzir a remodelação cardíaca reversa.^9^

Dados do estudo *Comparison of Pre- and Post-discharge Initiation of LCZ696 Therapy in HFrEF Patients After an Acute Decompensation Event* (TRANSITION - Comparação de Início do Tratamento com LCZ696 em Pacientes com ICFER Após um Evento de Descompensação Aguda Antes e Após a Alta), demonstrou que o início do INRA como primeira linha de terapia para pacientes com reincidência de ICFER não alterou o índice de adoção de terapias de IC guiadas por diretrizes.^10^Além disso, nesse estudo, pacientes com reincidência de ICFER que iniciaram o INRA tiveram menos efeitos colaterais e índices mais baixos de descontinuidade do tratamento para pacientes com ICFER anterior.^10^ Da mesma forma nos pacientes com reincidência de ICFER, o INRA levou a uma diminuição mais rápida e maior de biomarcadores cardíacos, tais como o NT-proBNP e a troponina-T de alta sensibilidade, e índices mais baixos de IC e nova hospitalização global em comparação a pacientes com ICFER prévio.^10^Por último, um estudo prévio demonstrou que o INRA tinha uma boa relação custo-benefício comparado com o enalapril no tratamento da ICFER, do ponto de vista da saúde pública do Reino Unido, da Dinamarca e da Colômbia.^11^

Conforme analisado nesta carta, evidências anteriores corroboram a hipótese de que o INRA melhora a qualidade de vida geral e reduz o risco de mortalidade cardiovascular, hospitalização por IC, e NT-proBNP em pacientes com ICFER. Além disso, o INRA leva à melhoria do status de saúde, e à remodelação cardíaca reversa, e não altera a adoção de terapias guiadas por diretrizes no caso de ICFER. Entretanto, alguns autores criticam alguns aspectos do estudo PARADIGM-HF, incluindo sua dose alvo de enalapril (10 mg duas vezes ao dia),^4^ enquanto as diretrizes de IC da *European Society of Cardiology* (Sociedade Europeia de Cardiologia) e da Sociedade Brasileira de Cardiologia propõem uma dose alvo máxima tolerada. No entanto, a dose estabelecida como meta pelo estudo acompanhou as diretrizes do *American College of Cardiology* (Colégio Americano de Sociologia), com pacientes tendo atingido um bom nível de dose mediana, semelhante à de estudos randomizados anteriores. Outra questão é o fato de que o estudo PARADIGM-HF investigou a eficiência de uma dose de INRA de 100 a 200 mg, enquanto a eficiência de doses mais baixas, tais como 50 mg, que pode ser o máximo tolerado por alguns pacientes, ainda precisa ser testada.^4^

Embora sejam necessários futuros estudos para chegar a uma conclusão mais certa sobre a adoção do INRA como primeira linha de terapia para a ICFER, em nosso ponto de vista, os benefícios mencionados acima são um forte argumento para a adoção do sacubitril-valsartana como primeira linha de terapia da ICFER, em vez de IECA, na falta de um histórico de angioedema ou hipotensão significativa.

## Quais são os possíveis benefícios e riscos de se adotar SGLT2i como primeira linha de terapia da ICFER?

O estudo DAPA-HF comparou a dapagliflozina, um SGLT2i, com placebo em pacientes com ICFER nas classes II, III, e IV, com ou sem diabetes.^2^ Nesse estudo, pacientes tratados com dapagliflozina tiveram uma redução de 26% no risco de morte cardiovascular ou piora da IC, em comparação com o cuidado padrão simples, com um NNT = 21.^2^ Uma análise exploratória do DAPA-HF também confirmou a melhoria do resultado primário, independentemente do status do paciente de diabetes.^12^ Em relação à segurança, a frequência dos eventos adversos foi semelhante entre os grupos que utilizaram dapagliflozina e placebo.^3^ Devido à ação diurética do SGLT2i, foram levantadas dúvidas sobre o uso seguro desses fármacos em pacientes com ICFER tratados com diuréticos de alça e ARM.^13^ Entretanto, uma subanálise publicada recentemente do estudo DAPA-HF demonstrou que a melhoria dos sintomas e a tolerância ao tratamento não foram diferentes entre os subgrupos com uso de diuréticos diferentes.^13^ Estudos posteriores também investigaram se os benefícios da dapagliflozina nos resultados primários estavam relacionados à terapia de fundo da IC. Entretanto, um estudo anterior demonstrou que no estudo DAPA-HF, a dapagliflozina reduziu o resultado primário, independentemente da terapia de fundo.^14^Além disso, Solomon et al.^15^ demonstraram que a eficiência e a segurança da dapagliflozina foram semelhantes em pacientes que estavam fazendo uso de sacubitril/valsartana a pacientes que estavam usando o placebo no estudo DAPA-HF, o que sugere que a combinação desses agentes poderia reduzir ainda mais a ocorrência de mortalidade ou hospitalização em pacientes com ICFER.^15^Ademais, uma metanálise conduzida por Turgeon et al.,^16^ que incluiu dois estudos que analisaram mais de 4.000 pacientes com ICFER, demonstrou que a dapagliflozina melhorou significativamente a qualidade de vida do paciente, em comparação com o placebo.^16^

Recentemente, outro SGLT2i, a empagliflozina, alcançou seu desfecho primário no estudo EMPEROR-Reduced.^3^Nesse estudo, pacientes com ICFER tratados com empagliflozina tiveram uma redução de 25% no risco de morte cardiovascular ou piora da IC e redução de 30% no risco de hospitalização por IC.^3^ Além disso, o grupo tratado com empagliflozina tinha um índice mais lendo de declínio de taxa de filtração glomerular.^3^ Em relação à segurança, a infecção genital não complicada foi mais comum em pacientes tratados com empagliflozina.^3^ Dados do estudo EMPATROPISM também demonstraram que a empagliflozina melhorou significativamente os volumes do VE, a massa do VE, a função sistólica do VE, a capacidade funcional, e a qualidade de vida, quando comparado ao grupo que utiliza o placebo em pacientes não diabéticos com ICFER.^17^ Uma metanálise, que analisou dados dos estudos DAPA-HF e EMPEROR-Reduced, demonstrou que a dapagliflozina e a empagliflozina reduziram a morte global e cardiovascular, e apresentaram melhores resultados renais, confirmando ainda mais o papel importante do SGLT2i na ICFER.^18^ Além disso, esse estudo demonstrou que os benefícios do SGLT2i na ICFER não dependiam o status do paciente de diabetes, idade, sexo, o terapia com INRA.^18^ Em relação ao custo-benefício, a dapagliflozina demonstrou uma boa relação custo-benefício para pacientes com ICFER, sob a perspectiva da saúde pública do Reino Unido, Alemanha, e Espanha.^19^

Embora os SGLT2i reduzam o risco de morte cardiovascular e piora do IC e sejam bem tolerados, até hoje não há recomendações de seu uso nas diretrizes para IC. Em nosso ponto de vista, os SGLT2i podem ser seguramente instituídos como um novo pilar na primeira linha de terapia de pacientes com ICFER.

## Um novo padrão de terapia na ICFER

**Figura 1:**
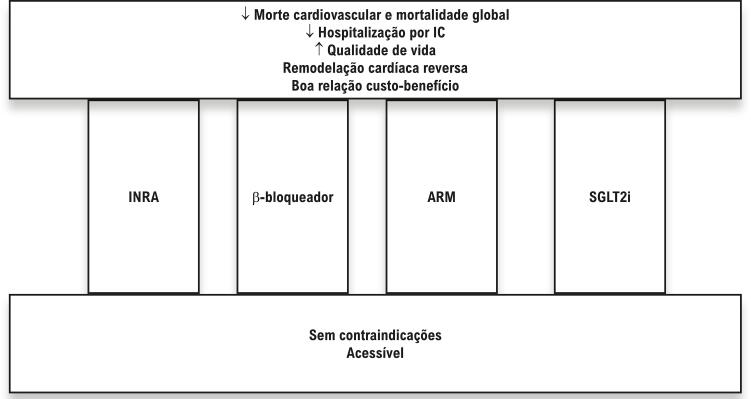
Proposta de nova primeira linha de terapia para pacientes com Insuficiência cardíaca com fração de ejeção reduzida. A barra inferior ilustra as condições necessárias para instituir uma combinação de inibidor da neprilisina e do receptor da angiotensina, betabloqueador, antagonista de receptor mineralocorticoide, e inibidores do cotransportador sódio-glicose 2. A barra superior ilustra os benefícios e a boa relação custo-benefício dessa combinação. INRA - inibidor da neprilisina e do receptor da angiotensina; IC: insuficiência cardíaca; ARM: antagonista de receptor mineralocorticoide; SGLT2i: inibidores do cotransportador sódio-glicose 2.

INRA e SGLT2i são fármacos bem tolerados e com boa relação custo-benefício que reduzem o risco de mortalidade, hospitalização, melhoria da qualidade de vida, e podem levar à remodelação cardíaca reversa, em comparação com a terapia convencional. Para ilustrar a importância dessas terapias, um estudo transversal comparou os efeitos de INRA, betabloqueadores, ARM, e SGLT2i (chamados de terapia abrangente) a IECA ou BRA e betabloqueador apenas (chamados de terapia convencional).^20^ Nesse estudo, pacientes tratados com terapia abrangente tiveram uma probabilidade 62% menor de sofrer morte cardiovascular ou hospitalização por IC.^20^ Além disso, a terapia abrangente foi superior na redução da morte cardiovascular, na hospitalização do IC, e da mortalidade global sozinha.^20^ Esse estudo também estimou que a terapia abrangente garantiu anos adicionais sem morte cardiovascular ou da primeira hospitalização por IC, e estendeu a sobrevida.^20^Uma proposta de uma nova terapia de primeira linha para ICFER está ilustrada na Figura 1.

## Conclusão

As atuais diretrizes do IC ainda não recomendam a proposta de primeira linha de terapia discutidas neste trabalho. Embora o custo de novos fármacos seja sempre uma questão importante na prescrição, especialmente em países menos desenvolvidos e em desenvolvimento, tais como o Brasil, em nosso ponto de vista, evidências convincentes analisadas neste trabalho corroboram a recomendação de INRA e SGLT2i como primeira linha de terapia em casos de ICFER. Dessa forma, diretrizes futuras para IC deveriam recomendar que uma combinação de INRA, betabloqueadores, ARM, e SGLT2i como o novo padrão de primeira linha de tratamento para ICFER em pacientes sem contraindicação para o uso desses medicamentos. Para garantir a adoção dessas novas terapias, médicos podem apresentar seus benefícios e sua relação custo-benefício aos pacientes. Além disso, órgãos de saúde públicos e convênios de saúde devem reconhecer as vantagens econômicas desses novos fármacos e desenvolver medidas para ajudar sua implementação.
