# Supplementary material for: Chegou a Hora de uma Nova Terapia Padrão para a Insuficiência Cardíaca com Fração de Ejeção Reduzida?
Source: Arq Bras Cardiol. 2021 Jul 15;117(1):149–52. [Article in Portuguese] doi: 10.36660/abc.20200983 (PMC8294725; doi:10.36660/abc.20200983)
Supplement: Translation

# Translation: Is It Time for a New Standard Therapy for Heart Failure with Reduced Ejection Fraction?

**Authors:** Eduardo Thadeu de Oliveira Correia, Letícia Mara dos Santos Barbetta, Evandro Tinoco Mesquita

**Affiliations:** 1 Brazil Hospital Universitário Antônio Pedro, Niterói, RJ - Brazil; 2 Brazil Complexo Hospitalar de Niterói, Niterói, RJ - Brazil

**Keywords:** Heart Failure, Stroke Volume, Drug Therapy, Mortality, Hospitalization, Ventricular Modeling, Neprilysin, Angiotensins

## Introduction

Heart failure (HF) with reduced ejection fraction (HFrEF) leads to mortality and impairment in one’s quality of life, and causes a major impact on the healthcare system. Despite trials showing the benefits of angiotensin receptor–neprilysin inhibitor (ARNI) over angiotensin-converting enzyme inhibitors (ACEi), and the superiority of sodium-glucose cotransporter-2 inhibitors (SGLT2i) over placebos, current guidelines still recommend ACEi, mineralocorticoid receptor antagonists (MRA), and beta-blockers as first-line therapy in HFrEF.^1 - 3^In this letter, we will discuss the potential benefits and risks of adopting ARNI and SGLT2i as first-line therapies in HFrEF.

## What are the potential benefits and risks of adopting ARNI as a first-line therapy in HFrEF?

In the PARADIGM-HF trial, HFrEF patients treated with ARNI had a significant reduction in the primary outcome of cardiovascular mortality or HF hospitalization (21.8% vs. 26.5%; number needed to treat (NNT) = 21) compared with enalapril.^1^Moreover, ARNI significantly reduced the all-cause mortality (17.0% vs. 19.8%; NNT = 36).^1^A sub-analysis of the PARADIGM-HF trial has also shown that ARNI significantly improved one’s quality of life compared with enalapril.^4^Regarding safety, sacubitril/valsartan led to higher proportions of hypotension and non-serious angioedema, but lower proportions of renal impairment, hyperkalemia, and cough compared with enalapril.^1^The benefits of ARNI over enalapril were further confirmed in patients with HFrEF hospitalized for acute decompensated HF in the PIONEER-HF trial, which showed a significant reduction in N-terminal pro–B-type natriuretic peptide (NT-proBNP) in patients treated with ARNI since week one.^5^A reduction in a composite of HF rehospitalization or cardiovascular death was also found to be significant in patients treated with ARNI in an exploratory analysis of the PIONEER-HF trial.^6^Finally, a sub-analysis of the PIONEER trial showed that ARNI was well-tolerated and superior to enalapril in improving clinical outcomes, regardless of ACE inhibitor, ARB treatment, or previous HF history.^7^

Real word data from the Change the Management of Patients with Heart Failure registry (CHAMP-HF) has also shown an association between ARNI therapy and early improvements in health status compared to patients not treated with ARNI.^8^In the EVALUATE-HF trial, which aimed to access if ARNI compared with enalapril improved central aortic stiffness and cardiac remodeling, ARNI led to a significant reduction in secondary echocardiographic end-points, suggesting that ARNI may induce reverse cardiac remodeling.^9^

Data from the Comparison of Pre- and Post-discharge Initiation of LCZ696 Therapy in HFrEF Patients After an Acute Decompensation Event (TRANSITION) study, showed that first-line initiation of ARNI in *de novo* HFrEF did not change the adoption rate of guideline-directed HF therapies.^10^Moreover, in this study, patients with *de novo* HFrEF that initiated ARNI had fewer side-effects and lower rates of treatment discontinuations compared to patients with prior HFrEF.^10^Moreover, in *de novo* HFrEF, ARNI led to a faster and greater decrease in cardiac biomarkers, such as NT-proBNP and high-sensitivity troponin-T, and lower rates of HF and all-cause rehospitalization when compared to patients with prior HFrEF.^10^Finally, a previous study showed that ARNI was cost-effective when compared with enalapril in HFrEF from the healthcare perspective of the United Kingdom, Denmark, and Colombia.^11^

As reviewed in this letter, previous evidence supports that ARNI improves overall quality of life and reduces the risk of cardiovascular mortality, HF hospitalization, and NT-proBNP in patients with HFrEF. Furthermore, ARNI leads to an improvement in health status and to reverse cardiac remodeling, and does not change the adoption of guideline-directed therapies in HFrEF. However, some authors criticize some aspects of the PARADIGM-HF trial, including its target dose of enalapril (10 mg twice daily),^4^whereas the European Society of Cardiology and the Brazilian Society of Cardiology HF guidelines support a maximum tolerated target dose. Nonetheless, the dose targeted by the trial followed the American College of Cardiology guidelines, with patients having achieved a good level of median dose, similar to previous randomized trials. Another issue is that the PARADIGM-HF trial investigated the effectiveness of an ARNI dose of 100-200 mg, while the effectiveness of lower doses, such as 50 mg, which may be the maximum tolerated dose for some patients, has yet to be tested.^4^

Although future studies are needed to bring a strong conclusion for the adoption of ARNI as a first-line therapy in HFrEF, in our view, the benefits mentioned above make a strong case for the adoption of sacubitril-valsartan as a first-line therapy in HFrEF instead of ACEi, in the absence of a history of angioedema or significant hypotension.

## What are the potential benefits and risks of adopting SGLT2i as a first-line therapy in HFrEF?

The DAPA-HF trial compared dapagliflozin, an SGLT2i, with a placebo in class II, III, and IV HFrEF patients with or without diabetes.^2^In this study, patients treated with dapagliflozin had a 26% reduction in the risk of cardiovascular death or worsening HF, compared with standard care alone, with an NNT = 21.^2^An exploratory analysis of the DAPA-HF has also confirmed the improvement of primary outcome, regardless of the patient’s status of diabetes.^12^Regarding safety, the frequency of adverse events was similar between the dapagliflozin and placebo group.^3^Because of the diuretic action of SGLT2i, concerns were raised about whether these drugs could be safely used in HFrEF patients treated with loop diuretics and MRA.^13^However, a recently published sub-analysis of the DAPA-HF trial showed that symptom improvement and treatment toleration did not differ across subgroups with different diuretic usage.^13^Further studies also investigated if the benefit of dapagliflozin in primary outcomes was related to background HF therapy. However, a previous study showed that in the DAPA-HF trial, dapagliflozin reduced the primary outcome regardless of background therapy.^14^Moreover, Solomon et al.^15^showed that the effectiveness and safety of dapagliflozin were similar in patients who were taking sacubitril/valsartan with patients who were taking the placebo in the DAPA-HF trial, which suggests that the combination of these agents could further reduce the occurrence of mortality or hospitalization in patients with HFrEF.^15^Furthermore, a meta-analysis of Turgeon et al.,^16^which included two trials that analyzed over 4,000 patients with HFrEF, showed that dapagliflozin significantly improved the patient’s quality of life when compared with the placebo.^16^

Recently, another SGLT2i, empagliflozin, met its primary endpoint in the EMPEROR-Reduced trial.^3^In this trial, HFrEF patients treated with empagliflozin had a 25% reduction in the risk of cardiovascular death or hospitalization for HF and a 30% reduction in the risk of hospitalization for HF.^3^Furthermore, the empagliflozin-treated group had a slower rate of decline in the glomerular filtration rate.^3^Regarding safety, uncomplicated genital infection was more common in patients treated with empagliflozin.^3^Data from the EMPATROPISM trial has also shown that empagliflozin significantly improves LV volumes, LV mass, LV systolic function, functional capacity, and quality of life when compared with the placebo in non-diabetic patients with HFrEF.^17^A meta-analysis, that analyzed data from DAPA-HF and EMPEROR-Reduced trials, showed that dapagliflozin and empagliflozin reduced all-cause and cardiovascular death, and improved renal outcomes, further confirming the important role of SGLT2i in HFrEF.^18^In addition, this study showed that the benefits of SGLT2i in HFrEF were independent of the patient’s status of diabetes, age, sex, or ARNI therapy.^18^Regarding cost-effectiveness, dapagliflozin proved to be cost-effective for patients with HFrEF based on the UK, German, and Spanish healthcare perspective.^19^

Although the SGLT2i reduce the risk of cardiovascular death and worsening HF, and are well tolerated, to date, there are still no recommendations of its use in HF guidelines. In our view, SGLT2i can be safely instituted as a new pillar of first-line therapy in HFrEF patients.

## A new standard therapy in HFrEF

ARNI and SGLT2i are well-tolerated and cost-effective drugs that reduce the risk of mortality and hospitalization, improve the quality of life, and may lead to reverse cardiac remodeling when compared to conventional therapy. To illustrate the importance of these therapies, a cross-trial study compared the effects of ARNI, beta-blocker, MRA, and SGLT2i (called comprehensive therapy) with ACEi or ARB and beta-blocker only (called conventional therapy).^20^In this study, patients treated with comprehensive therapy were 62% less likely to experience cardiovascular death or HF hospitalization.^20^Furthermore, comprehensive therapy was superior in reducing cardiovascular death, HF hospitalization, and all-cause mortality alone.^20^This study also estimated that comprehensive therapy provided additional years free from cardiovascular death or first hospitalization for HF and extended survival.^20^A proposal of a new first-line therapy for patients with HFrEF is illustrated in Figure 1 .

**Figure 1:** - Proposal of a new first-line therapy for patients with Heart Failure with Reduced Ejection Fraction. The bottom bar illustrates the conditions needed to institute a combination of angiotensin receptor–neprilysin inhibitor, beta-blocker, mineralocorticoid receptor antagonists, and sodium-glucose cotransporter-2 inhibitors. The upper bar illustrates the benefits and cost-effectiveness of this combination. ARNI - angiotensin receptor–neprilysin inhibitor; HF: heart failure; MRA: mineralocorticoid receptor antagonists; SGLT2i: sodium-glucose cotransporter-2 inhibitors.

## Conclusion

Current HF guidelines still do not recommend the first-line therapy proposal discussed in this paper. Although the cost of new drugs is always an important issue in prescriptions, mainly in less developed and developing countries, such as Brazil, in our view, compelling evidence reviewed in this paper supports the recommendation of ARNI and SGLT2i as first-line therapies in HFrEF. Hence, future HF guidelines should endorse a combination of ARNI, beta-blocker, MRA, and SGLT2i as the new standard first-line therapy for HFrEF in patients without contraindications to these medications. To ensure the adoption of these new therapies, physicians can present their benefits and cost-effectiveness to patients. Beyond that, public health agencies and private insurance plans should recognize the cost-effectiveness of these new drugs and develop measures to help their implementation.
